# Author Correction: Oxidative post-translational modification of EXECUTER1 is required for singlet oxygen sensing in plastids

**DOI:** 10.1038/s41467-026-77712-9

**Published:** 2026-09-21

**Authors:** Vivek Dogra, Mingyue Li, Somesh Singh, Mengping Li, Chanhong Kim

**Affiliations:** 1 Shanghai Center for Plant Stress Biology and Center of Excellence in Molecular Plant Sciences, Chinese Academy of Sciences, Shanghai, China; 2 University of the Chinese Academy of Sciences, Beijing, China

Correction to: *Nature Communications* https://doi.org/10.1038/s41467-019-10760-6, published online 27 June 2019

In the version of the article initially published, due to a figure preparation mistake, in Figs. 4b and 5b, the confocal images of protochlorophyllide fluorescence were mistakenly duplicated. Due to the age of the article, the figures cannot be updated directly. The correct figures are available as Supplementary Information accompanying this amendment. This notice serves to correct the article.

## Supplementary information

Correct Figs. 4, 5

