# Supplementary material for: Oxidative post-translational modification of EXECUTER1 is required for singlet oxygen sensing in plastids
Source: Nat Commun. 2019 Jun 27;10:2834. doi: 10.1038/s41467-019-10760-6 (PMC6597547; doi:10.1038/s41467-019-10760-6)
Supplement: Supplementary file 1 — Supplementary Information [file 41467_2019_10760_MOESM1_ESM.pdf]

# Supplementary Information

# Oxidative post-translational modification of EXECUTER1 is required for singlet oxygen sensing in plastids

Dogra, Li, Singh et al.

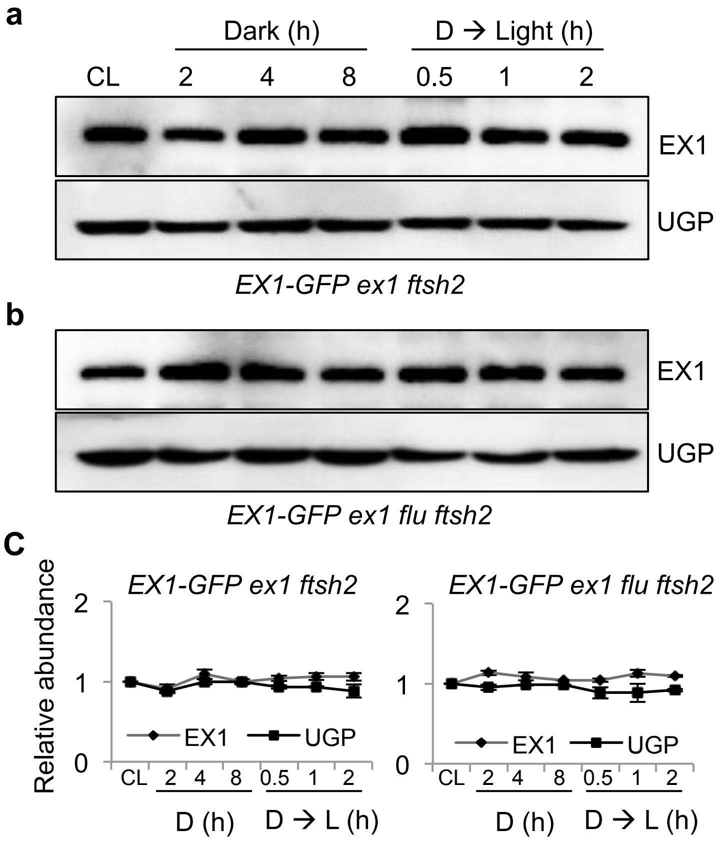


**Supplementary Fig. 1** Light- and $^1O_2$-dependent EX1 degradation is FtsH protease dependent. CL-grown 5-d-old transgenic seedlings of **(a)** *ftsh2 ex1* and **(b)** *ftsh2 ex1 flu* expressing *EX1-GFP* under the control of the 35S promoter were transferred to the dark (for 2, 4, 8 h) and then re-exposed to light (for 0.5, 1, 2 h) at the light intensity of 100 $\mu mol \cdot m^{-2} \cdot s^{-1}$. Total protein was extracted and analyzed by western blot. Cytosolic UDP-glucose pyrophosphorylase (UGP) was used to show equal loading. EX1-GFP and UGPase were detected using antibodies against GFP, and UGP, respectively. (**c**) The levels of EX1-GFP in the dark or after re-exposing to light were compared to its abundances under CL conditions. Average intensity values of the protein bands were calculated using AzureSpot software v14.0 (AZURE). Data represents mean of three biological repeats. Error bars show standard error of the mean.

**a**

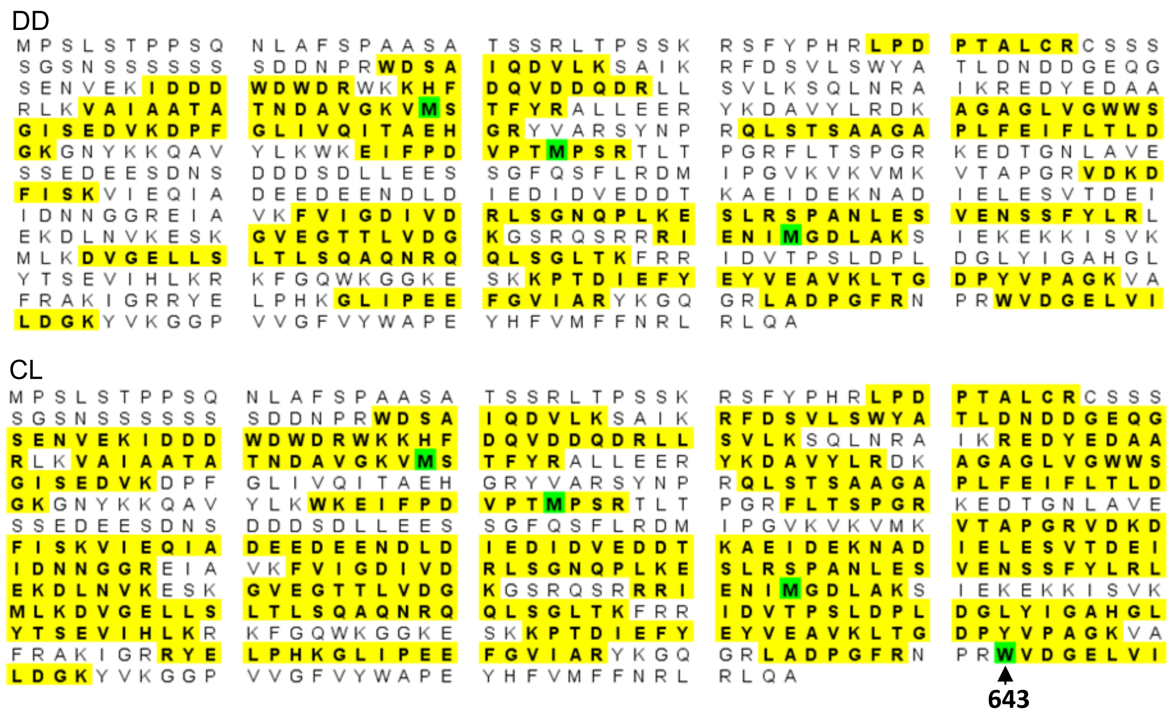


| Sample | Protein identification Probability (%) | Protein in total spectra (%) | Exclusive unique peptide count | Exclusive unique spectrum count | Total spectrum count | Coverage (%) |
|---|---|---|---|---|---|---|
| DD | 100 | 0.22 | 26 | 37 | 53 | 40 |
| CL | 100 | 0.32 | 34 | 59 | 98 | **53** |

**b**

MPSLSTPPSQNLAFSPAASATSSRLTPSSKRSFYPHRLPDPTALCRCSSSSGSNSSSSSSSDDNPR**W**DSA 70
IQDVLKSAIKRFDSVLS**W**YATLDNDDGEQGSENVEKIDDD**W**D**W**DR**W**KKHFDQVDDQDRLLSVLKSQLNRA 140
IKREDYEDAARLKVAIAATATNDAVGKVMSTFYRALLEERYKDAVYLRDKAGAGLVG**WW**SGISEDVKDPF 210
GLIVQITAEHGRYVARSYNPRQLSTSAAGAPLFEIFLTLDGKGNYKKQAVYLK**W**KEIFPDVPTMPSRTLT 280
PGRFLTSPGRKEDTGNLAVESSEDEESDNSDDDSDLLEESSGFQSFLRDMIPGVKVKVMKVTAPGRVDKD 350
FISKVIEQIADEEDEENDLDIEDIDVEDDTKAEIDEKNADIELESVTDEIIDNNGGREIAVKFVIGDIVD 420
RLSGNQPLKESLRSPANLESVENSSFYLRLEKDLNVKESKGVEGTTLVDGKGSRQSRRRIENIMGDLAKS 490
IEKEKKISVKMLKDVGELLSLTLSQAQNRQQLSGLTKFRRIDVTPSLDPLDGLYIGAHGLYTSEVIHLKR 560
KFGQ**W**KGGKESKKPTDIEFYEYVEAVKLTGDPYVPAGKVAFRAKIGRRYELPHKGLIPEEFGVIARYKGQ 630
GRLADPGFRNPR**W**VDGELVILDGKYVKGGPVVGFVY**W**APEYHFVMFFNRLRLQA 684

643

**Supplementary Fig. 2** MS analysis showed a significant peptide coverage for EX1. **a** Peptide coverage of EX1 protein from dark-(DD) and light-(CL) grown seedlings as visualized in Scaffold viewer (Scaffold 4.0.7, Proteome Software Inc.). Peptides identified by tandem-MS analysis are shown in yellow. Oxidized Met (M) and Trp (W) residues are highlighted in green. Protein identification parameters are enlisted at the bottom. **b** EX1 protein contains 11 Trp residues (highlighted in bold), and all were covered in MS analysis (shown in grey background). Trp643 undergoing $^1O_2$-induced oxidation is highlighted in red. UVR B/C and DUF3506 (SOS) domains are underlined.

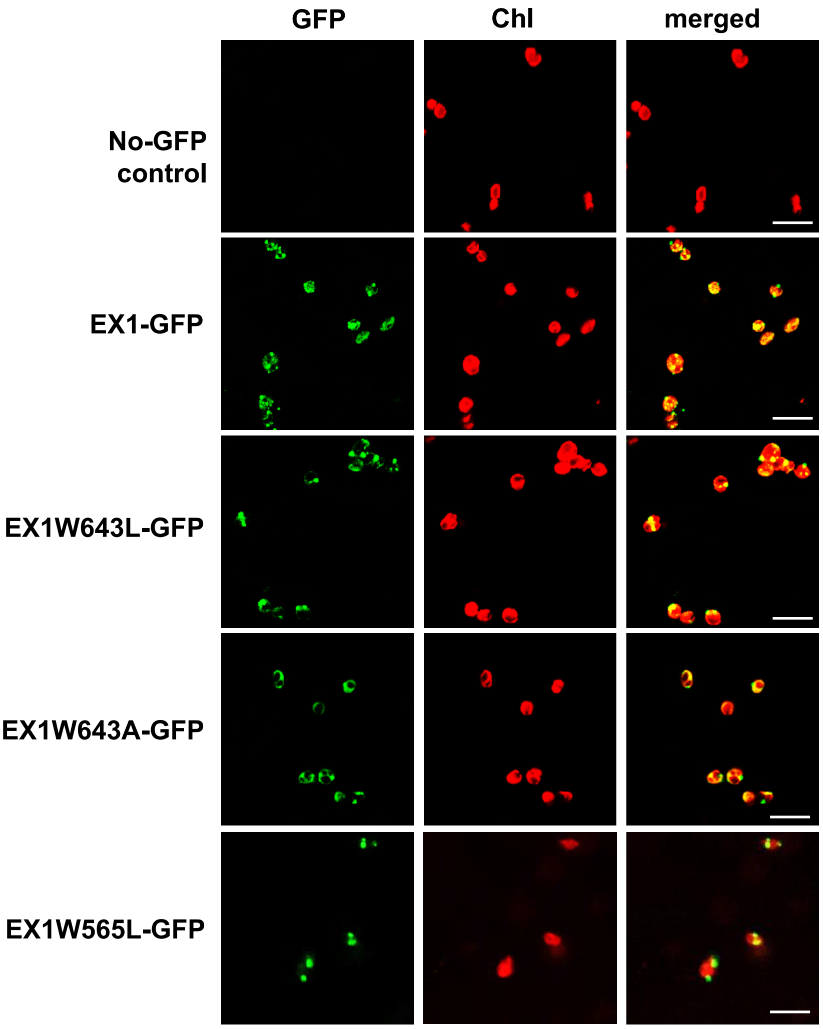


**Supplementary Fig. 3** EX1 proteins carrying Trp643 substitutions are targeted to chloroplasts. Confocal images showing the localization of EX1-GFP, EX1W643A-GFP, EX1W643L-GFP, and EX1W565L-GFP in the chloroplasts of *N. benthamiana* leaves. Healthy leaves of 3-weeks-old *N. benthamiana* were infiltrated with Agrobacterium carrying 35S::*EX1-GFP*, 35S::*EX1W643A-GFP*, 35S::*EX1W643L-GFP,* and 35S*::EX1W565L-GFP*. The GFP signal was observed under confocal laser scanning microscope. Leaves without infiltration were used as negative controls.

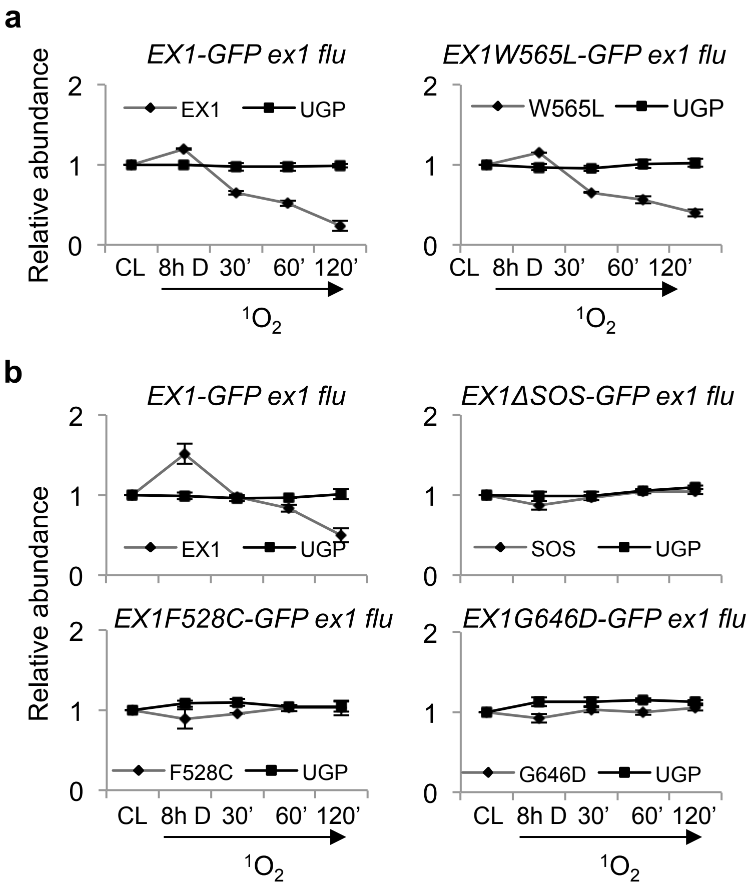


**Supplementary Fig. 4** Relative abundances of intact and modified EX1 proteins upon $^1O_2$ burst. The steady state levels of intact and modified EX1-GFP proteins upon dark- or after re-exposing to light were compared to their abundances under CL conditions as shown in (**a**) figures 4g and (**b**) 5e. The abundance of UGP was shown as equal loading control. Average intensity values of the protein bands were calculated using AzureSpot software v14.0 (AZURE). Data represents mean of three biological repeats. Errors bars show standard error of the mean.

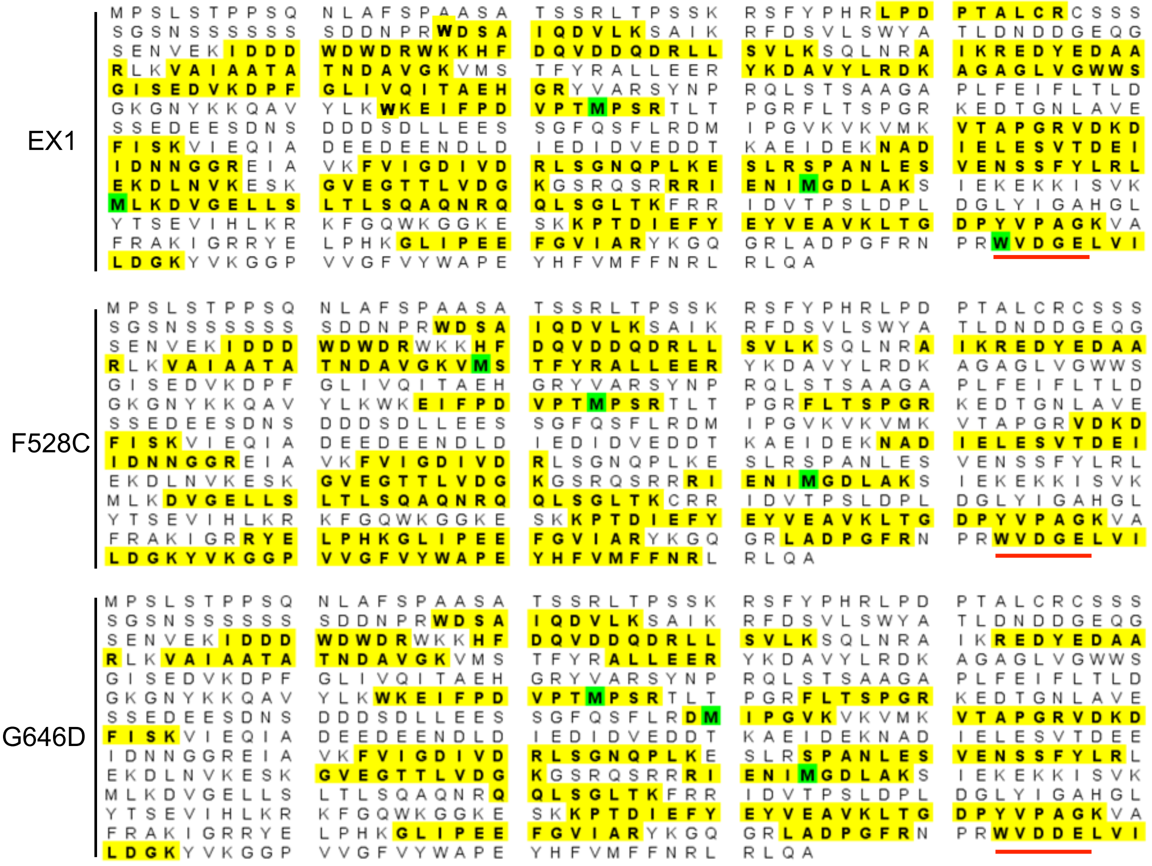


| Protein | Protein identification Probability (%) | Total spectrum count | Coverage (%) |
|---|---|---|---|
| EX1 | 100 | 53 | 40 |
| F528C | 100 | 30 | 39 |
| G646D | 100 | 29 | 37 |

**Supplementary Fig. 5** Oxi-PTM analysis revealed Trp643 undergoes oxidation in EX1-GFP but not in EX1F528C-GFP and EX1G646D-GFP proteins. For oxidation analysis, 5-d-old transgenic seedlings of *EX1-GFP ex1 flu, EX1F528C-GFP ex1 flu*, and *EX1G646D-GFP ex1 flu* initially grown under CL condition were transferred in the dark, kept for 2 h, and re-exposed to light for 10 min. Total protein was extracted and the immune-reactive EX1-GFP proteins were enriched by using magnetic agarose beads conjugated with GFP antibody. Each protein sample was then subjected to PTM analysis. Peptide coverage of EX1 protein from EX1-GFP, EX1F528C-GFP and EX1G646D-GFP samples was visualized in Scaffold viewer. Peptides identified by tandem-MS analysis are shown in yellow. Oxidized Met (M) and Trp (W) residues are highlighted in green.

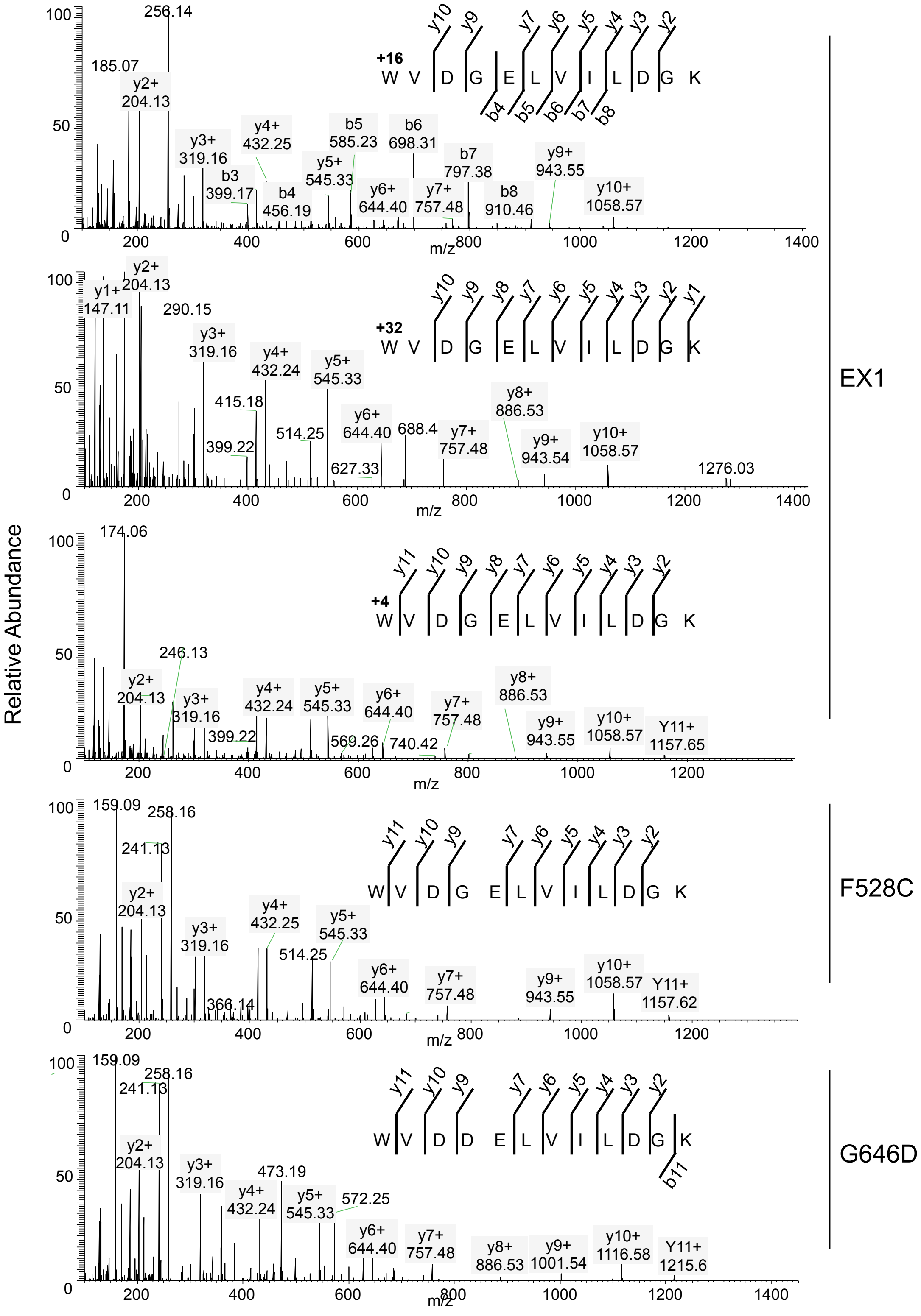


**Supplementary Fig. 6** MS spectra of peptides carrying Trp643 in native and modified EX1 proteins. All three oxidized variants of Trp643 in peptide [643]WVDGELVILDGK[654] were detected in EX1-GFP. However, Trp643 remains unoxidized in peptides [643]WVDGELVILDGK[654] in EX1F528C-GFP and [643]WVDDELVILDGK[654] in EX1G646D-GFP.

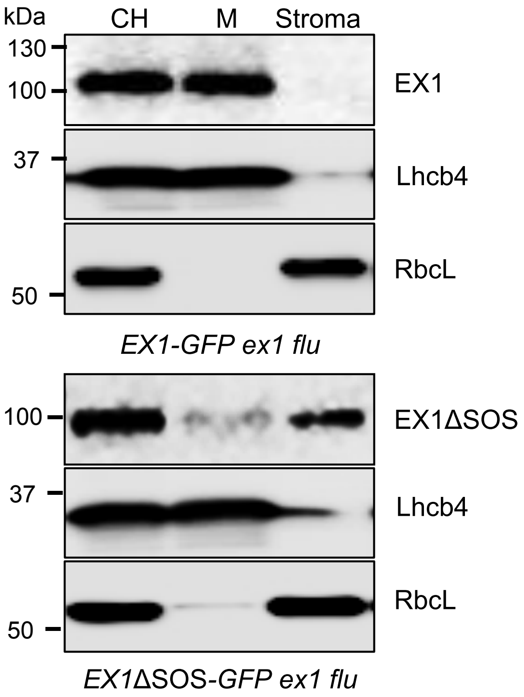


**Supplementary Fig. 7** SOS domain-deleted EX1 is unable to attach to the thylakoid membrane which is indispensible for perceiving $^1O_2$. EX1-GFP proteins reside in the thylakoid membrane whereas the majority of the EX1ΔSOS-GFP were found in the stroma. Chloroplast (CH) isolated from 3-weeks-old plants of *EX1-GFP ex1 flu* and *EX1ΔSOS-GFP ex1 flu* were lysed and separated by centrifugation into membrane (M), and stroma fractions. Proteins were solubilized, separated by SDS-PAGE, and analyzed on western blots using specific antibodies against GFP, Lhcb4, and Rubisco large subunit (RbcL). Lhcb4 and RbcL serve as membrane and stroma controls, respectively.

**Supplementary Table 1:** MS-based PTM analysis identified various Trp oxidation products at Trp643 in peptide $^{643}$WVDGELVILDGK$^{654}$.

| Oxidation status | Mass shift | Observed mass (m/z) | Charge state | Actual mass (Da) | Error (ppm) | Probability (%) | Mascot ion score |
|---|---|---|---|---|---|---|---|
| No | 0 | 672.37 | 2+ | 1342.72 | 0.0026 | 100 | 62.2 |
| Mono (OIA) | +16 | 680.36 | 2+ | 1358.71 | 1.4 | 100 | 43.1 |
| Di (NFK) | +32 | 688.36 | 2+ | 1374.71 | 4.7 | 100 | 63.8 |
| Di* (KYN) | +4 | 674.36 | 2+ | 1346.71 | -0.0011 | 100 | 64.7 |

OIA: oxindolylalanine, NFK: *N*-formylkynurenine, KYN: Kynurenine

**Supplementary Table 2:** MS-based PTM analysis identified $^1O_2$-induced oxidation at Trp643 side chains in EX1 but not in EX1 proteins carrying F528C and G646D substitutions.

| Protein | Sequence | Residue modified | Mass shift | Observed mass (m/z) | Charge state | Actual mass (Da) | Error (ppm) | Probability (%) |
|---|---|---|---|---|---|---|---|---|
| EX1 | $^{643}W^{ox}VDGELVILDGK^{654}$ | Trp643 | +16 | 680.36 | 2+ | 1358.71 | 3.0 | 98 |
| EX1 | $^{643}W^{ox}VDGELVILDGK^{654}$ | Trp643 | +32 | 688.36 | 2+ | 1374.70 | -0.68 | 100 |
| EX1 | $^{643}W^{ox}VDGELVILDGK^{654}$ | Trp643 | +4 | 674.36 | 2+ | 1346.71 | 1.2 | 100 |
| EX1F528C | $^{643}WVDGELVILDGK^{654}$ | No | 0 | 672.36 | 2+ | 1342.71 | -0.91 | 100 |
| EX1G646D | $^{643}WVDDELVILDGK^{654}$ | No | 0 | 701.37 | 2+ | 1400.37 | -0.59 | 100 |

**Supplementary Table 3:** List of primers used in this study.

| Gene ID | Gene name | Primer sequence (5' to 3') | Primer length | Product size (bps) | Used for |
|---|---|---|---|---|---|
| AT3G18780 | *ACT2* | F: TATGTATGTCGCCATCCAA | 19 | 76 | qRT-PCR |
| | | R: ACCAGAATCCAGCACAATA | 19 | | |
| AT2G38470 | *WRKY33* | F: GAAACAAATGGTGGGAATGG | 20 | 217 | |
| | | R: TGTCGTGTGATGCTCTCTCC | 20 | | |
| AT3G56710 | *SIB1* | F: CGACTTTTCTCACCACGACA | 20 | 235 | |
| | | R: TCGGAGGAAGGGGAATAGAT | 20 | | |
| AT2G41180 | *WRKY40* | F: GTCAAGACGCCGCTGATCTA | 20 | 166 | |
| | | R: GCCGACATCTGAGGAGCATT | 20 | | |
| AT3G18780 | *ACT2* | F: CTGGGATTTGTAAAGCAGCTG | 21 | 265 | RT-PCR |
| | | R: TCAAGTTCCATGGAGCAAAAG | 21 | | |
| AT4G33630 | *EX1-GFP* | F: ATGCCTTCCTTATCCACCCCG | 20 | 2159 | |
| | | R: CCATTAACATCACCATCTAAT | 21 | | |
| AT4G33630 | *EX1* | F: GGGGACAAGTTTGTACAAAAAAGCAGGCTATGCCTT CCTTATCCACCCCG | 20 | EX1 | Cloning and AA substitution and domain deletion [1st PCR: F & R1, F1 and R; 2nd PCR: F + R using mixture of purified primary products] |
| | | R: GGGGACCACTTTGTACAAGAAAGCTGGGTCGGCTTG AAGCCTCAGGCGGTT | 21 | | |
| | | F1: CGTAATCCGAGAGCGGTAGATGGCGAG | 27 | 643A | |
| | | R1: CTCGCCATCTACCGCTCTCGGATTACG | 27 | | |
| | | F1: CGTAATCCGAGATTGGTAGATGGCGAG | 27 | 643L | |
| | | R1: CTCGCCATCTACCAATCTCGGATTACG | 27 | | |
| | | F1: CAAATTTGGCCAGTTGAAAGGTGG | 24 | 565L | |
| | | R1: CCACCTTTCAACTGGCCAAATTTG | 24 | | |
| | | F1: CTCAAAACCGTCAACAGGGCAAGTATGTAAAAGG | 34 | ΔSOS | |
| | | R1: CCTTTTACATACTTGCCCTGTTGACGGTTTTGAG | 34 | | |
