# Supplementary material for: Oxidative post-translational modification of EXECUTER1 is required for singlet oxygen sensing in plastids
Source: Nat Commun. 2019 Jun 27;10:2834. doi: 10.1038/s41467-019-10760-6 (PMC6597547; doi:10.1038/s41467-019-10760-6)
Supplement: Supplementary file 3 — Description of Additional Supplementary Files [file 41467_2019_10760_MOESM3_ESM.pdf]

Description of Additional Supplementary Files

File Name: Supplementary Data 1
Description: List of the proteins found to be associated with EX1 in dark- and light-grown seedlings. IP-enriched samples were subjected to mass spectrometry followed by Mascot database searches. Proteins were considered co-precipitated, if found in at least three biological replicates by mass spectrometry.
