# Supplementary material for: Author Correction: Oxidative post-translational modification of EXECUTER1 is required for singlet oxygen sensing in plastids
Source: Nat Commun. 2026 Sep 21;17:10021. doi: 10.1038/s41467-026-77712-9 (PMC13594157; doi:10.1038/s41467-026-77712-9)

Fig. 4
with corrections in panel 4b
(replaced images *W643L* and *W565L*)

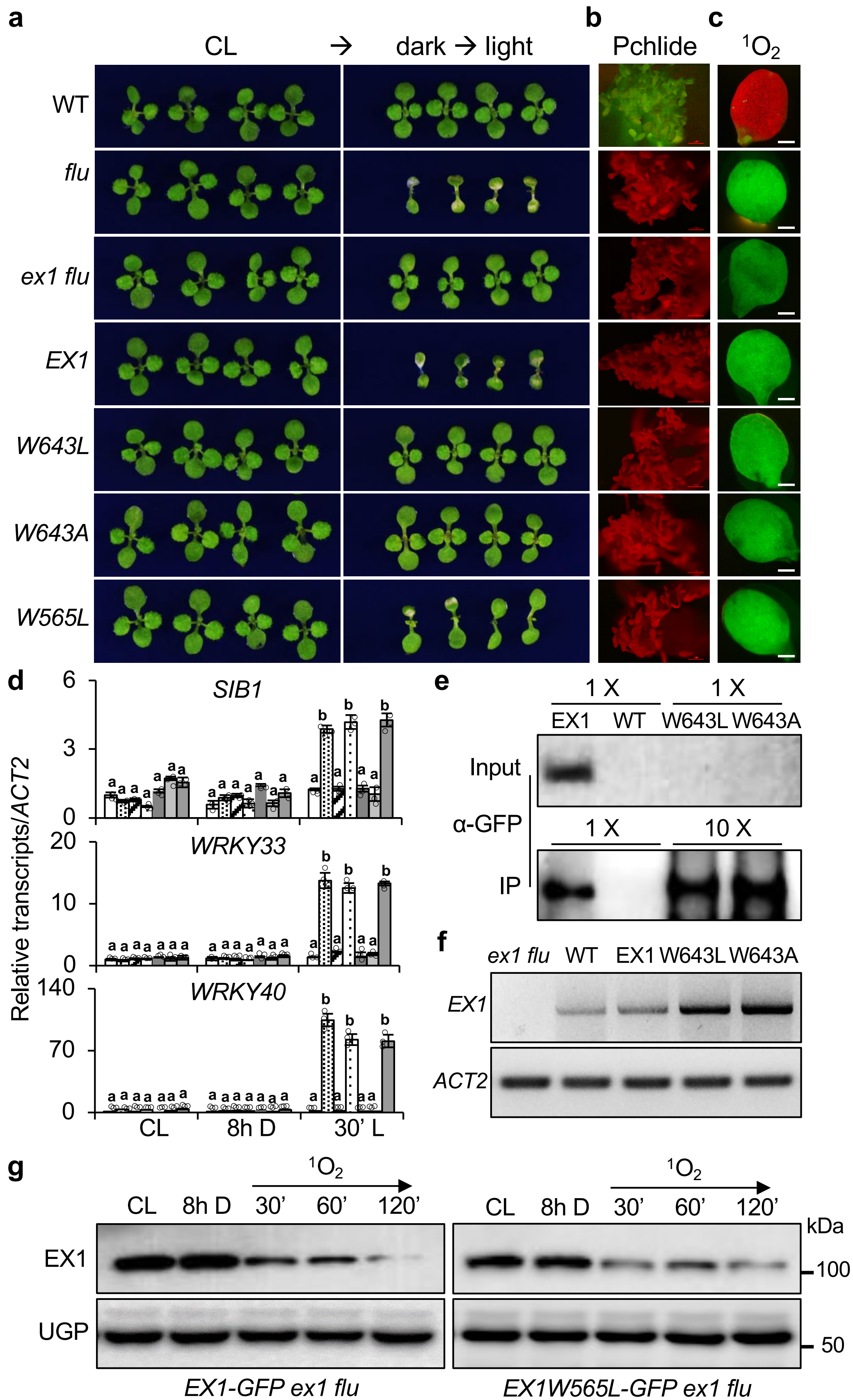

Fig. 5
with corrections in 5b panel
(replaced images *flu* and G*646D*)

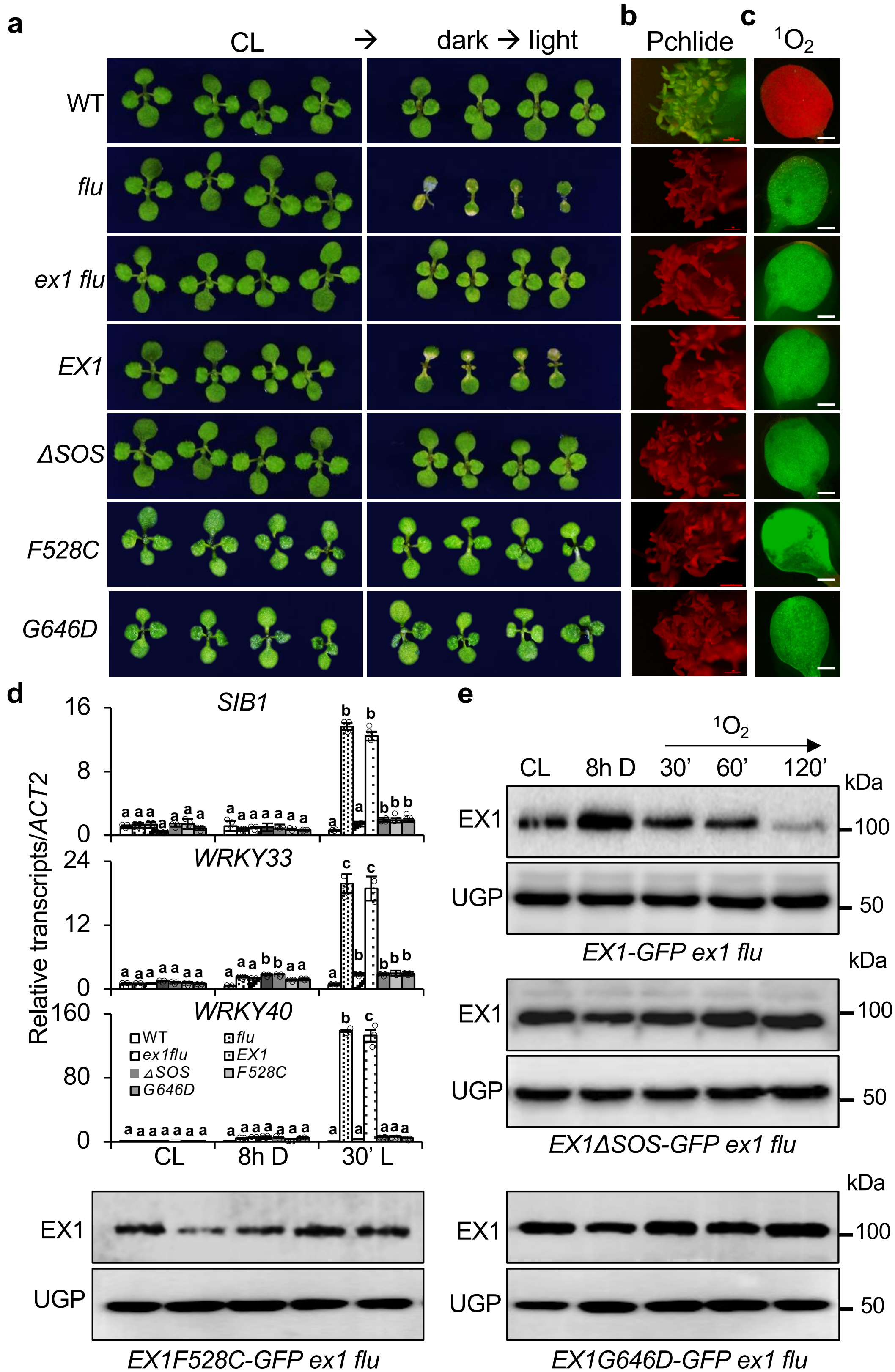

Supplement: Supplementary file 1 [file 41467_2026_77712_MOESM1_ESM.pdf]
